# Gamevar.f90: a software package for calculating individual gametic diversity

**DOI:** 10.1186/s12859-020-3417-x

**Published:** 2020-03-06

**Authors:** Daniel Jordan de Abreu Santos, John B. Cole, George E. Liu, Paul M. VanRaden, Li Ma

**Affiliations:** 1 Department of Animal and Avian Sciences, University of Maryland, College Park, MD 20742 USA; 2 Henry A. Wallace Beltsville Agricultural Research Center, Animal Genomics and Improvement Laboratory, ARS, USDA, Beltsville, MD 20705-2350 USA

**Keywords:** Mendelian sampling, Gamete, Recombination, Complex trait

## Abstract

**Background:**

Traditional selection in livestock and crops focuses on additive genetic values or breeding values of the individuals. While traditional selection utilizes variation between individuals, differences between gametes within individuals have been less frequently exploited in selection programs. With the successful implementation of genomic selection in livestock and crops, estimation and selection for gametic variation is becoming possible.

**Results:**

The gamevar.f90 software is designed to estimate individual-level variance of genetic values of gametes for complex traits in large populations. The software estimates the (co)variances of gametic diversity as well as other diversity parameters that are useful for selection programs and mating designs. The calculation is carried out chromosome by chromosome and can be easily parallelized. The gamevar.f90 program is written in Fortran with efficient computing algorithms in a user-friendly software package with easily-handled input and output files. Finally, we applied the program to estimate gametic variance for hundreds of bulls for lifetime net merit, productive life, and livability. The RPTA (relative predicted transmitting ability), assuming a future selection intensity (*i*_*f*_) of 1.5, showed larger variance than GEBV/2, indicating that greater future genetic gains can be obtained using an index that includes gametic variances. We also used the relative coefficient of variation to estimate with 95% confidence the sample sizes required to observe 90% variability of the progeny for lifetime net merit (or to allow at maximum 10% of change in the EBV predicted from progeny data).

**Conclusions:**

Collectively, we develop an efficient computer program package, gamevar.f90, for estimating gametic variance for large numbers of individuals. The novel information on gametic variation will be useful in future animal and crop breeding programs.

## Background

Traditionally, selective breeding programs and mating designs are based only on the estimated breeding values (EBVs) of individuals, aiming for the genetic improvement of additive merit. The EBV represents the sum of additive effects of all genes. The individual’s EBV is an average of its parents’ EBVs plus an independent effect from Mendelian sampling caused by random recombination and separation of homologous chromosomes [1]. Mendelian sampling variability differs across individuals and can be estimated as a function of the binomial transmission probabilities of DNA variants from individuals to gametes and their genetic effects [2]. Therefore, the variability generated by Mendelian sampling and meiotic recombination can be assessed from genomic data. Initially, Selgeke et al., [3] estimated the variance of the EBVs within groups of offspring by simulating virtual gametes of individuals. Subsequently, Bonk et al. [4] proposed an explicit formula to obtain this variation of the within-family EBVs. More recently, based on quantitative trait loci (QTL) effects in the gametes, Santos et al. [2] proposed the variance of the gametic diversity (${\upsigma}_{\mathrm{gamete}}^2$). Assuming a large number of QTL are transmitted from an individual to its gametes, the genetic values of all possible gametes will follow a normal distribution with variance equal to the ${\upsigma}_{\mathrm{gamete}}^2$, and the sum of variance of two matting individuals is equal to the variance of future progeny (also known as Mendelian Sampling variance) [2]. These authors then evaluated the predictability by genomic models in a dataset containing only markers or with markers and QTLs, obtaining medium to high predictability. When the solution of the genomic models is used, the ${\upsigma}_{\mathrm{gamete}}^2$ is partly like what was proposed by Bonk et al. [3], with differences only in the central probability matrix. Despite ${\upsigma}_{\mathrm{gamete}}^2$ represents the capture of the variation of the effects of QTLs on gametes, in the specific case, it is also equivalent to the variance of the gametes breeding values, whose average is equal to EBV/2.

The gametic variance ${\upsigma}_{\mathrm{gamete}}^2$ is a useful tool for identifying individuals that are more likely than their peers to produce gametes and thus progeny with extreme breeding values. In addition, gametic variance can be combined with breeding value into a new selection index, RPTA (relative predicted transmitting ability), which selects for genetic diversity to improve genetic gain in the long term [2]. The RPTA is a measure with biological interpretation, whose value represents the expected difference (on average) of the selected gametes, in relation to the genetic base of the population, when a certain selection intensity is applied to all gametes of an individual. The selection with RPTA is projected in the variation of gametes (as the proportion of selected gametes or selection intensity); however, in practice, the real selection is realized in the variation of the future progeny. Based on this, Bijma et al. [5] recommended an index with linear approximation with the within-family standard deviation. However, this linearization assumes that the ${\upsigma}_{\mathrm{gamete}}^2$ of the sire and dam is the same, making this index less accurate for the selection of the future progeny. This assumption of equality can be avoided with our software that can easily estimate the ${\upsigma}_{\mathrm{gamete}}^2$ of the animals to be selected and mated.

The ${\upsigma}_{\mathrm{gamete}}^2$ can be used to estimate the coefficient of relative variation (CRV) that measures the variability in the percentage of additive genetic values transmitted from an individual to its gametes (EBV/2), which is useful in breeding and progeny testing programs to estimate the optimal number of progeny needed to realize the expected gametic variability [2]. This parameter can be used and interpreted as the traditional coefficient of variation, which, however, has no limitation for negative values and zeros in the denominator. Santos et al. [2] proposed the CRV that allows assessing the variation associated with EBV. In addition, the CRV may be more suitable than the traditional coefficient of variation (it allows values greater than 100%) to estimate sample sizes needed to realize certain levels of gametic variance [6].

In this study, we implemented our recently developed method into the gamevar.f90 software that efficiently estimates gametic variance for complex traits in large populations. Basically, gamevar.f90 calculates individual-level genetic statistics per chromosome such as EBVs, (co)variances of gametic diversity, and coefficients of relative variation, as well other genetic components useful to estimate the relative selection index (such as RPTA) for designing selective mating programs and progeny tests.

## Implementation

### Method

The gamavar.90 program estimates the (co)variance of all possible gametic values that can be generated from an individual genome and meiosis process using data on phased genotype, allelic substitution effect, and recombination rate between variants. Since only the heterozygous loci of an individual will contribute to ${\upsigma}_{\mathrm{gamete}}^2$, the variance of two biallelic loci, *j* and *k*, of an individual *i*, with the true allele substitution effect α_j_ and α_k_, can be calculated from the variance of a binomial distribution as ${\upsigma}_{\left[\mathrm{j}+\mathrm{k}\right]}^2={\upsigma}_{\mathrm{j}}^2+{\upsigma}_{\mathrm{k}}^2+2{\upsigma}_{\mathrm{j}\mathrm{k}}$, where ${\upsigma}_{\mathrm{j}}^2= npq{\alpha}_j^2,{\upsigma}_{\mathrm{k}}^2= npq{\alpha}_k^2$, σ_ij_ = n(*p*_*jk*_ − *p*_*j*_*p*_*k*_)α_j_*α*_*k*_, and *p* = q = 0.5 and *n* = 1. Thus, the total variance is computed across all *N* heterozygous loci for trait *x* as ${\upsigma}_{\mathrm{Xgamete}}^2=\left[{\hat{\alpha}}_{x_1}\kern0.5em \dots \kern0.5em {\hat{\alpha}}_{x_N}\right]P\left[{\hat{\alpha}}_{x_1}\kern0.5em \dots \kern0.5em {\hat{\alpha}}_{x_n}\right]^{\prime }$ and the covariance between the traits *x* and *y* can be computed using the same matrix P (as in Santos et al. [2]), and the allele substitution effect of the two traits as in (Bonk et al. [4]), as $$ {\sigma}_{XYgamete}=\left[{\hat{\alpha}}_{x_1}\kern0.5em \dots \kern0.5em {\hat{\alpha}}_{x_N}\right]P\left[\begin{array}{ccc}{\hat{\alpha}}_{y1}& \dots & {\hat{\alpha}}_{y_N}\end{array}\right]^{\prime } $$, where $\hat{\alpha}$ is the allele substitution effect estimated with genomic model. The (co)variance matrix of the Mendelian transmission probabilities, **P,** with only the heterozygous loci can be represented as $$ P=\left[\begin{array}{ccc}0.25& \dots & {al}_{1N}\left(-\frac{cM_{1N}}{200}+0.25\right)\\ {}\vdots & \ddots & \vdots \\ {}{al}_{N1}\left(-\frac{cM_{N1}}{200}+0.25\right)& \dots & 0.25\end{array}\right] $$, where al_jk_ is a phase indicator for loci *j* and *k*, with value 1 when both loci have the reference allele on the same chromosome and − 1 otherwise; cM_jk_ is the genetic distance between the 2 loci (in centimorgans). Loci with genetic distances greater than 50 cM on the same chromosome, are assumed to be independent. If the recombination rates between the SNP markers are directly used instead of cM, the off-diagonal elements of the **P** matrix will be ${P}_{jk}={al}_{jk}\left(-\frac{rate_{jk}}{2}+0.25\right)$ when the recombination rate is < 0.5; and *P*_*jk*_ = 0 when the rate is ≥0.5.

The gamevar.f90 software also calculates the chromosome-level statistic HOM = $\sum \limits_i^{NHom}{\alpha}_i^2$ (sum of squared effects of the homozygous loci from an individual) and coefficient of relative variation (CRV), ${CRV}_i=\frac{\sigma_{gamete}}{\sqrt{0.5{\sum}_i^{NHom}{\alpha}_i^2+{\upsigma}_{\mathrm{gamete}}^2}}$, as described by Santos et al. [2]. The statistics ${\upsigma}_{\mathrm{gamete}}^2$ and CRV include all chromosomes used in the calculation of genomic breeding values. Gamevar.f90 calculates these statistics for each of the chromosomes separately. Math for the sex chromosomes could differ by sex of parent and progeny but we treated all chromosomes as autosomes. The total statistics can be obtained as a simple total across the chromosomes. Details on these variability statistics and algorithms have been described in Santos et al. [2].

The gamevar.f90 program directly uses allele effects of the markers estimated from existing genomic evaluations. Since the allele effects have been estimated, gamevar.f90 can also calculate the genomic breeding values (it computes by chromosomes) according to Meuwissen et al. [7] as *M*[*α*_1_…*α*_*N*_]′, where M is a matrix of genotypes coded in − 1,0 and 1 for aa, Aa and AA, with rows corresponding to individuals and column to markers.

### Input and output files

A parameter file is required to run gamevar.f90. The parameter file provides some user-specified options, including file names. The program automatically performs an initial check of the parameters from the input file, such as the options defined by users, initial data descriptions, warnings, stoppings, cases of incorrect inputs, and output messages. Parameters are annotated in more details in the user’s manual (Additional File 1; https://github.com/djordand2008/gamevar.f90). Gamevar.f90 also requires some pre-processed files as input, such as allelic substitution effects and phased genotypes, as well as the chromosome information with recombination rate/genetic distance between markers. The program can optionally produce up to five easily-handled output files in text format for the (co)variance of gametic diversity, EBV, CRV and HOM by individuals. To reduce memory required by the program, output files are written during the analyses so that memory can be reused. In additional to the manual, ready-to-run example files are also provided in the package.

### Efficiency

The software is written in Fortran with the intrinsic library (Additional File 2). Executable files are currently available for the Linux platform (Additional File 3). It is free software with open-access code that is portable to other operating systems for compiling. The standard compilers for Fortran 90 and 95, such as gfortran, are recommended for use. In an example run, the computing time for analyzing eight traits (lifetime net merit, productive live, somatic cell score, daughter pregnancy rate, heifer conception rate, cow conception rate, livability, and early calving) with 4340 Markers on chromosome 1 and 100 bulls was around 4 to 5 min or less than 3 s per individual on an Intel Xeon X7560 server, running at 2.27GHz with 660GB RAM. A maximum of 0.15GB of RAM was used for the example run.

## Results

**Fig. 1:**
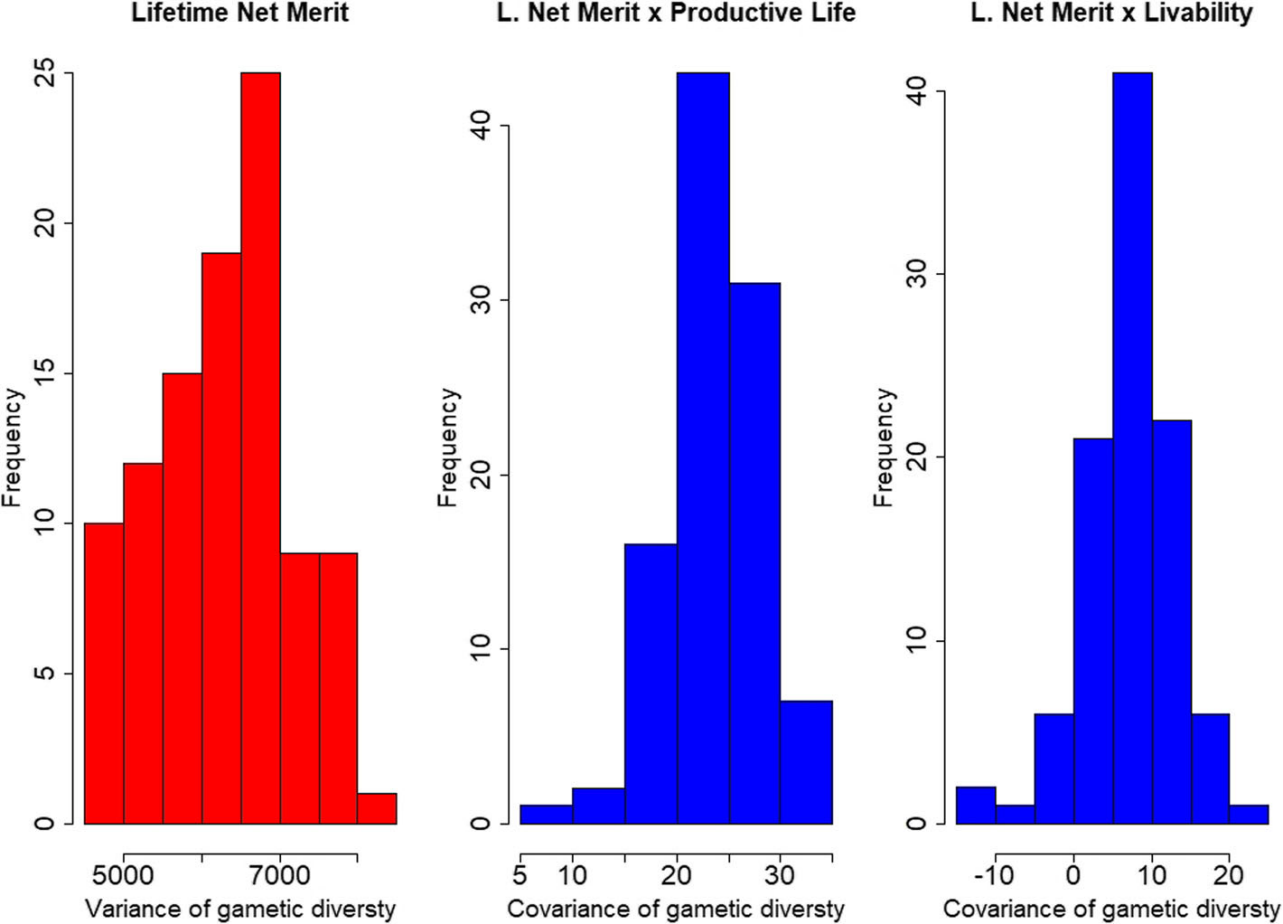
Histogram of variance of gametic diversity for lifetime net merit (left) and covariance of gametic diversity between lifetime net merit and productive life (middle) and livability (right) using the top 100 bulls for lifetime net merit

**Fig. 2:**
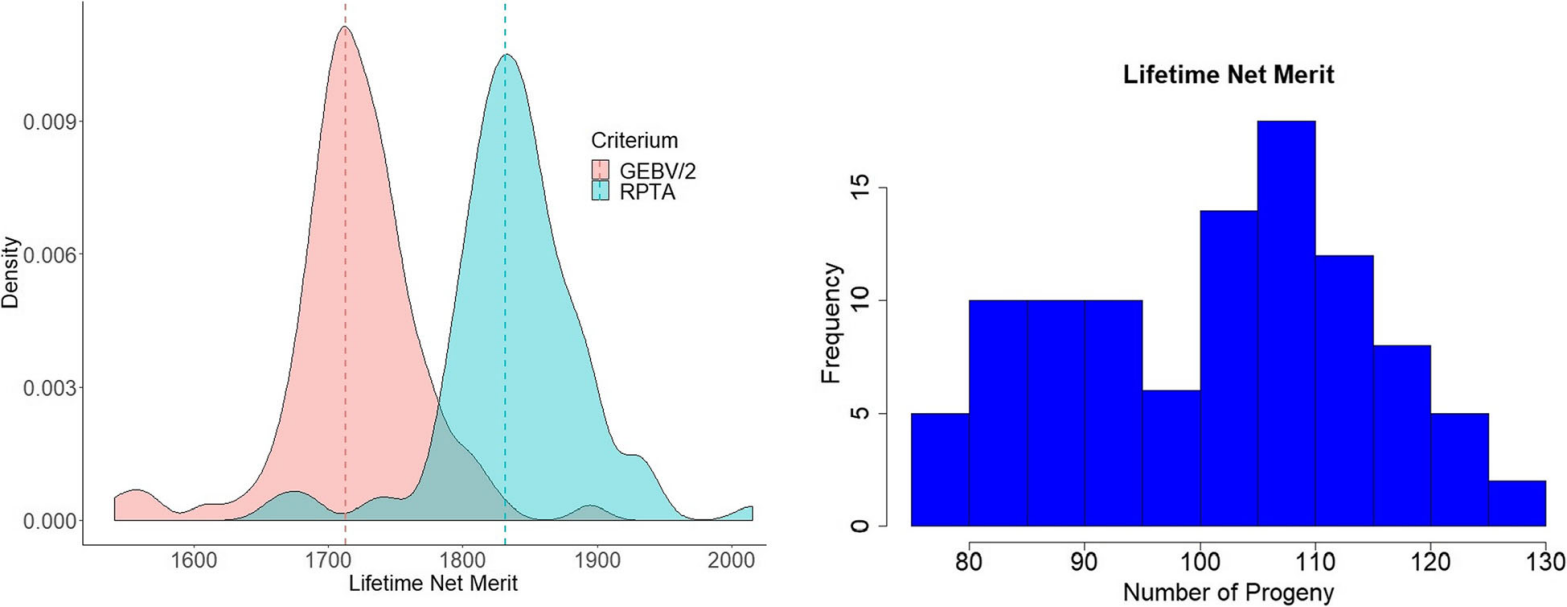
Density plot of estimated genomic breeding value (GEBV/2) and relative predicted transmitting ability (RPTA) assuming future selection intensity of 1.5 for lifetime net merit (left) and histogram of the number of progeny required to realize 90% of gametic variance (or to allow at maximum 10% of change in the EBV predicted using only progeny data) in the future progeny for the top 100 bulls of lifetime net merit (right)

Using gamevar.f90, we estimated gametic variance and other statistics of lifetime net merit for the 100 top Holstein bulls in the U.S. dairy industry. There is a considerable amount of variation in gametic diversity across the top 100 bulls (Fig. 1), which indicates the potential of applying gametic selection to the dairy cattle population. The covariances of gametic diversity were all positive between lifetime net merit and productive life, indicating that gametic selection in lifetime net merit could improve productive life. However, nine bulls showed negative covariances of lifetime net merit with livability, meaning that not all top bulls for lifetime net merit can improve livability in the population. In such cases, we can use gametic selection to identify bulls which will improve both traits simultaneously. The RPTA (*GEBV*_*i*_/2 + *σ*_*gamete* _ *i*_ ∗ *i*_*f*_), assuming a future (gametic) selection intensity (*i*_*f*_) of 1.5, for the 100 best bulls for lifetime net merit, showed greater variance and greater density beyond the center of its distribution compared with the GEBV/2, indicating that greater future genetic gains (represented by the means of the criteria) can be obtained with this index (density plot in Fig. 2). Evidently, greater gains can be achieved if a small number of bulls with extreme values (the left side of the density plot) were selected within this group (by increasing the selection intensity). Using the relative coefficient of variation of lifetime net merit, we estimated with 95% confidence the number of progeny required to observe 90% variability in the progeny (or to allow at maximum 10% of change in the EBV predicted using only progeny data, such as a progeny test). The number of progeny was calculated based on Santos et al. [2], as $n=\frac{(1.96)^2X{\left({CRV}_i\right)}^2}{(0.1)^2}$. Thus, the histogram in the second part of Fig. 2 shows that the number expected to realize a reasonable percentage of variation in gametes, ranged from 80 to 130. This number can be especially important for planning matings considering accuracy and cost for progeny production.

## Conclusions

Gametic diversity is an important source of genetic variation to be explored in selective breeding programs, which can be beneficial for both improving genetic gains and maintaining genetic diversity over the long term. Gamevar.f90 is a user-friendly tool for estimating the variance of gametic diversity in large-scale genomic data of complex traits in livestock and crop populations. Gamevar.f90 uses efficient algorithms, is easy to use, and takes advantage of multiple processors to achieve good computing performance in general. The output from gamevar.f90 will be useful for improving selection strategies, mating designs, and progeny tests.

## Availability and requirements

**Project name:** gamevar.f90.

**Project home page (**github page**):** https://github.com/djordand2008/gamevar.f90 **Operating system(s):** Linux and Unix **Programming language:** Fortran **Other requirements:** None **License:** GPL-v3 **Any restrictions to use by non-academics:** No (free software).

## Supplementary information

**Additional file 1.** Manual of Gamevar.f90. A word document describing the manual of the software.

**Additional file 2.** Source code of Gamevar.f90. An Fortran source code file for gamevar.f90.

**Additional file 3.** Executable file of Gamevar.f90. An executable file for linux system.

## Data Availability

The software, manual, and example data are available at Github page: https://github.com/djordand2008/gamevar.f90

## Abbreviations

CRV: Coefficient of relative variation
EBV: Estimated breeding value
GEBV: Estimated genomic breeding value
QTL: Quantitative trait locus
RPTA: Relative predicted transmitting ability
SNP: Single nucleotide polymorphism
