# Supplementary material for: Gamevar.f90: a software package for calculating individual gametic diversity
Source: BMC Bioinformatics. 2020 Mar 6;21:100. doi: 10.1186/s12859-020-3417-x (PMC7060627; doi:10.1186/s12859-020-3417-x)
Supplement: Supplementary file 1 — Additional file 1. Manual of Gamevar.f90. A word document describing the manual of the software. [file 12859_2020_3417_MOESM1_ESM.docx]

**gamevar.f90**

**A small and practical software for (co)variance of gametic diversity and other components of the coefficient of relative variation of the genetic additive values estimation**

Daniel Jordan de Abreu Santos^1^, John B. Cole^2^, Paul M. VanRaden^2^ and Li Ma1

^1^Department of Animal and Avian Sciences, University of Maryland, College Park, 20742

^2^Henry A. Wallace Beltsville Agricultural Research Center, Animal Genomics and Improvement Laboratory, ARS, USDA, Beltsville, MD 20705-2350

July 12, 2019

License

gamevar.f90 was developed by University of Maryland and partnership with USDA in order to benefit the U.S. dairy cattle genetic evaluations and has been partially financed by the USDA National Institute of Food and Agriculture (Washington, DC), the US-Israel Binational Agricultural Research and Development Fund, and the São Paulo Research Foundation (FAPESP). This is a free Fortran software: you may use, redistribute, modify and improve the code under the terms of version 3 of the GNU General Public License. Please report any bugs to [daniel_jordan2008@hotmail.com](mailto:daniel_jordan2008@hotmail.com).

Contents

**1 INTRODUCTION ...................................................................................................................................3**

**2 COMPILING……....................................................................................................................................4**

**3 INPUT FILE FORMATS ………………………………………..................................................................5**

**4 RUN .......................................................................................................................................................8**

**5 OUTPUTS…….......................................................................................................................................9**

**1 - Introduction**

gamevar.90 is software for estimation of individual genetic variability parameters that are useful for selection strategies, mating decisions, and progeny-testing designs. Genomic selection and mating programs for the improvement of additive genetic merit are based on the estimated breeding values (EBVs) of the individuals in the population. The EBV represents the sum of the average effects of the genes, which is independent of the variability of meiotic recombination events (i.e., separation of homologous chromosomes and crossover). Mendelian sampling variability differs across individuals and can be estimated as a function of the binomial transmission probabilities of DNA variants from individuals to gametes and their genetic effects. Assuming a large number of quantitative trait loci (QTL) are transmitted from an individual to its gametes, the genetic values of all possible gametes will follow a normal distribution with variance equal to the variance of gametic diversity ($\sigma_{\mathrm{gamete}}^{2}$). The standard deviation of gametic diversity can be combined with EBV to produce a new selection index, RPTA, which selects for genetic diversity to improve the genetic gain in the long term (Santos et al., 2019). Thus, gamavar.90 estimates the (co)variance caused by meiotic events using data on phased genotypes, allele substitution effects, and recombination rates between the variants from a chromosome.

gamevar.f90 also calculates per chromosome the component $\sum_{i}^{NHom} \alpha_{i}^{2}$ (HOM – sum of squared effects of the homozygous loci from an individual) and coefficient of relative variation (CRV) as described by Santos et al., (2019). The CRV measures the variability in the percentage of additive genetic values transmitted from an individual to its gametes. The useful statistics $\sigma_{\mathrm{gamete}}^{2}$ and CRV include all chromosomes used in the analysis to predict genomic breeding values (GEBV). gamevar.f90 processes each chromosome separately, which means that jobs may be easily parallelized. Overall statistics can be obtained in a second step by the user as simple sum by chromosome of the $\sigma_{\mathrm{gamete}}^{2}$ and the component $\sum_{i}^{NHom} \alpha_{i}^{2}$ of CRV, applying the formula $CRV=\frac{\sigma_{gamete}}{\sqrt{0.5\sum_{i}^{NHom} \alpha_{i}^{2}+\sigma_{\mathrm{gamete}}^{2}}}$.

gamevar.f90 does not calculate the RPTA, since it depends on the future intensity of selection and can also be adjusted by the average of individual percentage of variation in a future progeny group (given as function of number of progeny, the critical value associated with the degree of confidence, and the average of the population CRV).

**2 - Compiling**

The standard code in gamevar.f90 and its subroutines is written in the Fortran language without use of any additional libraries. Dynamic allocation is used throughout the code. The standard compilers for Fortran90 and 95, such as gfortran, are suitable and recommended. A version already compiled in Linux environment is also available, in case of compiling problems. An example using gfortran on a Unix terminal follows:

gfortran -o gamevar gamevar.f90

**3 - Input File Formats**

**3.1 – Parameter File**

A parameter file is required to run gamevar.f90. The parameter file provides some user-specific controls and the name of this file is determined by the user. The user can not add or delete any line in the parameter file. The number of traits specified must equal the number of allele effects columns. The name of the input files must be specified correctly bellow the “*_FILE” options. For the option GENETIC_DISTANCE_UNIT, the user must specify if the genetic distance between the variants is in centiMorgan (“morgans”) or recombination rate (“reco”). If the option is TRUE the software creates an output file with the variance and covariance of the gametic diversity, the component $\sum_{i}^{NHom} \alpha_{i}^{2}$, CRV or GEBV. If the option CRV is TRUE and variance of the gametic diversity is FALSE the software estimates the variance of gametic diversity internally but it does not output it, only the CRV result. An option with a desired name for the output files is also required. An example of a typical example of parameter file follows:

**
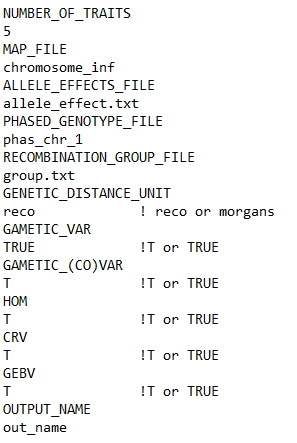
**

**3.2 - Chromosome information (MAP_FILE)**

This file is determined by the user and specified in the parameter file. The file has to follow the following format, without a header, and delimited by single space: Chromosome (CHR), SNP name (Name), SNP position (Position) and recombination rate/ genetic distance information (group1, group2 …) for the SNP. Only one chromosome can be specified in this file. The recombination rate/genetic distance information among the SNPs shall be as an accumulative function through the chromosome of SNP position, so that the number of recombination information in the file is equal to the number of SNP. At least one column with recombination information (for example if there is only a group) shall be provided. The maximum number of recombination group columns is given by the number of animals, in case the user has individual recombination rates. The number of columns for recombination groups shall be the same as specified in the recombination group file, and the order of the columns has to be the same as the first-in-time descending direction that the group labeled appears in that file.

**
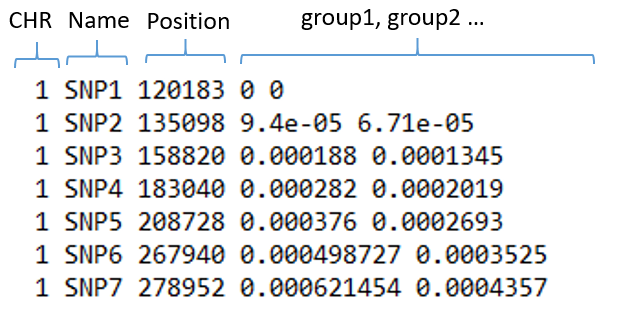
**

**3.3 – Recombination group (RECOMBINATION_GROUP_FILE)**

This is a reference to which information on recombination rate or genetic distance between the markers will be used per individual. The number of labeled group needs to match the number columns in chromosome information file. The order of the first appearance of the labeled groups shall be the same order as the columns with recombination rates/genetic distance in the chromosome information file. This file has the following format: Label of group (group) up to 10 characters and ID.

**
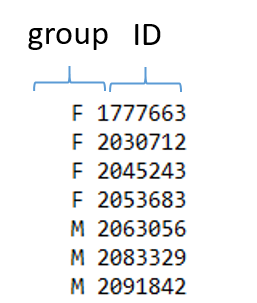
**

**3.4 – Allele effect information (ALLELE_EFFECTS_FILE)**

gamevar.f90 does not estimate the allele effects for the markers. The allele effects have to be estimated previously, using GBLUP or any differential shrinkage model (Santos et al., 2019). The file does not include a header, and contains marker solutions for each trait in the columns, and solutions for each SNP in the rows, delimited by a single space.

**
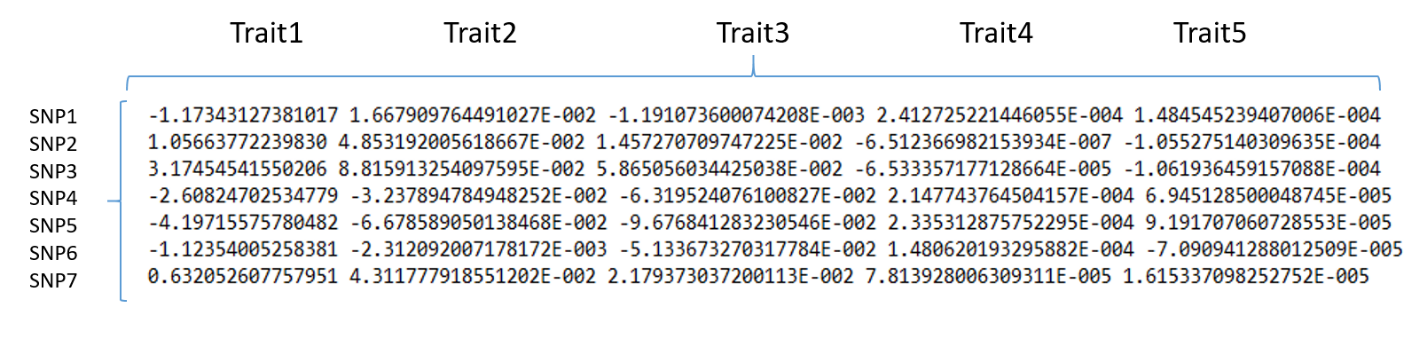
**

**3.5 – Phased genotype information (PHASED_GENOTYPE_FILE)**

The genotype data must already be phased, and the maternal and paternal gametic phases have to be ordered in rows tagged with same ID. The phased genotype file follow the format ID and haplotype phase delimited by a single space. The haplotype phase has no space between the alleles coded as 0 for A1 and 2 for A2. Since the genotype data was already phased, no missing allele/genotyped code is required, therefore accepted by gamevar.f90.

**
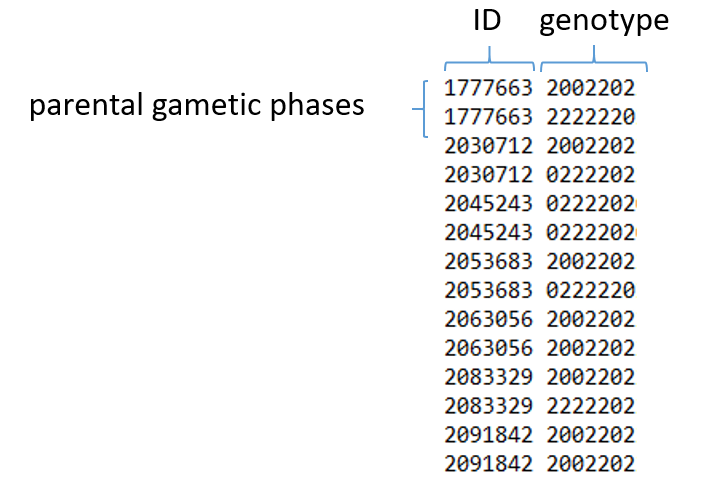
**

gamevar.f90 also reads genotypes in the PLINK PED file. To access this option just add at the end of the parameter file “OPTION PLINK”. The PED file must contain 6 columns with family ID, individual ID, Paternal ID, Maternal ID, Sex and Phenotype, besides other columns with the alleles encoded in 1 and 2. Remember that genotype data must be phased and without missing values. The .MAP file must be provided with chromosome, SNP name, recombination rate/ genetic distance information and SNP position, as well as the allele effects file. If the PLINK format is selected, only one recombination group will be used for all animals, whose information is in the third column of the MAP file, and the recombination group file is dispensable.**4 – Run**

gamevar.90 can be run on a terminal using a simple Linux command line with the parameter file name as an argument:

**./gamevar {parameter file name}**

The software runs only one chromosome per time. For run all chromosomes, or some groups of them, as well as split the running, the user can use a looping in Linux command like this:

**for i in {1..29}; do**

**echo "NUMBER_OF_TRAITS**

**5**

**MAP_FILE**

**reco_chr$i.txt**

**ALLELE_EFFECTS_FILE**

**alle_effec_$i**

**PHASED_GENOTYPE_FILE**

**phas_chr_$i**

**RECOMBINATION_GROUP_FILE**

**group.txt**

**GENETIC_DISTANCE_UNIT**

**reco**

**GAMETIC_VAR**

**TRUE**

**GAMETIC_(CO)VAR**

**T**

**CRV**

**T**

**GEBV**

**T**

**OUTPUT_NAME**

**output_chr_$i" > parameter.txt**

**echo parameter.txt |./gamevar**

**done**

**5 - Outputs**

**5.1 - Log**

Internal checks are printed on the screen, such as the options defined by user in the parameter file, initial data descriptions, warnings, stoppings, and output mensages, as follows:


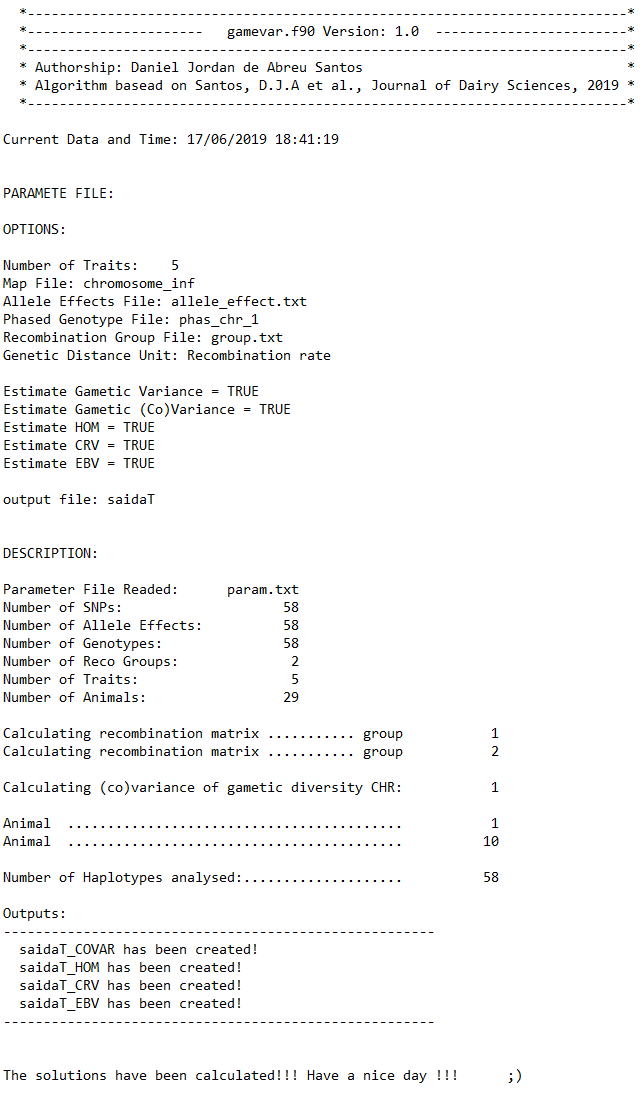


**5.2 - Output files**

Five output files can be created by gamevar.90:

{output_name}_EBV = solutions for EBVs;

{output_name}_VAR = output with variances of gametic diversity

{output_name}_COVAR = output with variance and covariance of gametic diversity

{output_name}_CRV = output with CRV;

{output_name}_HOM = output with $\sum_{i}^{NHom} 2\alpha_{i}^{2}$.

All of them have the solutions per individual in rows and header with ID and trait number, i.e.,TRAIT_{1,2,3..}. When the option GAMETIC_(CO)VAR isTRUE an extra number of column between the traits *i* and *j* with headers TRAIT_{i_1_,i_2_,i_3_..i_n_}_{j_1_,j_2_,j_3_...j_n_} are also written.

**Reference**

Santos, D.J.A., J.B. Cole, J. B., T.J. Lawlor Jr, VanRaden, P.M., Tonhati, H. and Ma, L. Variance of gametic diversity and its application in selection programs. Journal of Dairy Science, 102(6): 5279-5294, 2019.
